# The prevalence of orbital complications among children and adults with acute rhinosinusitis

**DOI:** 10.5935/1808-8694.20130131

**Published:** 2015-10-08

**Authors:** Mousa Victor Al-Madani, Ahmed Essa Khatatbeh, Rania Zaid Rawashdeh, Nemer Falah Al-Khtoum, Nabil Radwan Shawagfeh

**Affiliations:** a Dr. (Senior neuro-ophthalmology specialist department of Ophthalmology of King Hussein Medical Center of Jordanian Royal Medical Services); b Dr. (Senior ophthalmology specialist department of Ophthalmology of King Hussein Medical Center of Jordanian Royal Medical Services); c Dr. (ophthalmology specialist department of Ophthalmology of King Hussein Medical Center of Jordanian Royal Medical Services); d Dr. (Senior ENT specialist department of ENT of King Hussein Medical Center of Jordanian Royal Medical Services); e Dr. (Senior ENT specialist department of ENT of King Hussein Medical Center of Jordanian Royal Medical Services). Department of Ophthalmology of King Hussein Medical Center of Jordanian Royal Medical Services

**Keywords:** abscess, orbital cellulitis, sinusitis

## Abstract

Acute rhinosinusitis occurs commonly in children and adults being commoner in children. The prognosis is favorable in majority of cases.

**Objective:**

To investigate orbital complications in children and adult with sinusitis.

**Method:**

Patients attending ENT clinic with sinusitis from January 2010 until January 2012 were included. Patients were classified into two groups according to their age. First involved children aged less than 16 and second included adults older than 16 years. Clinical picture, sinus involved, management and outcome were compared.

**Results:**

The total number of patients were 616. Orbital complications were seen in 36 patients (5.8%). Twenty six patients (72.2%) were children (21 had preseptal and 5 had orbital cellulitis) and ten patients (27.8%) were adults (5 with preseptal, three with orbital cellulitis and 2 with abscess). The most common orbital complication was preseptal cellulitis (72.2%) followed by orbital cellulitis and abscess (22.2% and 5.6% respectively). The commonest sinus involved was ethmoidal in children and mixed sinus pathology in adults. The majority of patients responded to medical treatment.

**Conclusion:**

Orbital complications of sinusitis are commoner in children than adults and have favorable prognosis. Keywords: Sinusitis, cellulitis, preseptal, abscess.

## INTRODUCTION

Sinusitis is usually associated with concurrent rhinitis, rhinosinusitis is the preferred term for this condition1., 2.. It is often diagnosed clinically. Imaging may be needed when complications related to local spread of disease are suspected such as abscess formation or when patient fails to respond to medical treatment^3^.

Acute rhinosinusitis occurs commonly in children and adults being more common in children^4^. The prognosis is favorable without complications in the majority of cases^5^. Orbital complications are reported in 5% to 7% of patients1., 2., 6.. Orbital involvement is reported to occur more in children than in adults^7^. Ethmoidal sinus is the most commonly reported sinus to be involved in children. This is attributed to close relation between orbit and ethmoidal sinus in children with thin line separating between them8., 9.. In adults, the development of frontal sinus makes it a frequent cause of orbital involvement along with maxillary sinus^10^. Sphenoid sinus involvement is rare and may lead to optic nerve involvement^11^. There is controversy in literature regarding prognosis of orbital complications secondary to acute rhinosinusitis among children and adults and its response to treatment5., 6., 12.. Some studies showed that there is no difference regarding prognosis between children and adults while other showed that the prognosis is worse in adults.

We could not find a study in our area investigating the prognosis of orbital cellulitis among children and adults with acute rhinosinusitis. The aim of this study is to investigate the frequency of orbital complications in children and adult patients with sinusitis. Presentation, management and outcome were evaluated.

## METHOD

Patients attending ENT clinic diagnosed to have sinusitis during the period between January 2010 and January 2012 were enrolled in the study. Patients were selected from those who visited the ENT directly without referral from emergency room or other clinic. Only cases of acute sinusitis secondary to infection were included. The diagnosis was clinical in majority of cases. Acute rhinosinusitis was considered when patient complained of at least four weeks of purulent nasal drainage and obstruction associated with facial pain, pressure or fullness. CT scan was done when abscess was suspected or when patient failed to respond to medical treatment. Patients with orbital complications were referred to ophthalmology clinic. Ocular examination included best corrected visual acuity, anterior and posterior segment examination and extraocular motility examination.

Patients were classified into two groups according to their age. First group involved children aged less than 16 years and second group included adults older than 16 years. We chose the age of 16 years to separate between the two groups because patients younger than 16 years in our hospital are evaluated in pediatric clinics and those older than 16 are evaluated in adult clinics. Clinical picture, sinus involved, management and outcome were compared in two groups. Treatment protocol included topical and oral antibiotics for patients with periorbital cellulitis, broad spectrum intravenous antibiotics for patients with orbital cellulitis and abscess. Medications for pain relief and nasal decongestants were used in all patients.

Surgical drainage of abscess was considered when it failed to respond to medical treatment or if located posterior to the globe. *P*-value was used to determine statistical significance between the frequency of orbital complication in children and adults and considered significant when less than 0.05 was. An approval of the ethical committee was obtained before starting the study (number of protocol 272013).

## RESULTS

A total number of 616 patients were enrolled in the study. Orbital complications were seen in 36 patients (5.8%). Twenty six patients were children (72.2%) and ten patients (27.8%) were adults. The most common orbital complication was preseptal cellulitis seen in 72.2% of patients followed by orbital cellulitis and abscess in 22.2% and 5.6% respectively. In children, 21 out of 26 patients (80.8%) had preseptal cellulitis and 5 patients (19.2%) had orbital cellulitis. In adults, 50% of patients had preseptal cellulitis, 30% had orbital cellulitis and 20% had abscess. The frequency of orbital cellulitis and abscess in adults (30 % and 20% percent) were more statistically significant than in children (19.2% and zero), *p* value < 0.05 (Table 1).

**Table 1:** Distribution of patients according to orbital complications in children and adults.

Complication	Number of children	Number of adults	Total	p-value
Preseptal cellulitis	21	5	26	> 0.05
Orbital cellulitis	5	3	8	< 0.05
Abscess	-	2	2	< 0.05
Total	26	10	36	-

The most common sinus involved was ethmoidal sinus in children and mixed sinus pathology in adults. In children, sinuses involved were ethmoidal followed by mixed sinus pathology. Maxillary sinus was affected in four patients. In adults mixed sinus pathology was the most common (four patients). Frontal and maxillary sinuses were affected in three patients each. We did encounter any case of sphenoid sinus involvement in children and adults. The majority of patients responded to medical treatment. Ten patients were admitted and surgery was done for one patient (Table 2).

**Table 2:** Distribution of patients according to sinus involved.

Sinus of involvement	Number of children	Number of adults	Total
Frontal	-	3	3
Maxillary	4	3	7
Ethmoidal	16	-	16
Mixed	6	4	10
Total	26	10	36

## DISCUSSION

Acute infection the paranasal sinus is a rather frequent disease in children. On the contrary, the incidence of orbital complications secondary to sinusitis is low^2^. Orbital as well as intracranial complications of ethmoidal and maxillary sinusitis are most often encountered in childhood^13^. The majority of cases in childhood respond to medical treatment. On the other hand, severe morbidity and mortality occur if these complications are not appropriately treated. Such morbidity and mortality tend to occur in adults^4^.

Sinusitis is a clinical diagnosis in most cases. Radiology is rarely needed. It may be indicated in cases of ophthalmoplegia, proptosis, decreased visual acuity, failure of conservative treatment and when intraorbital and intracranial extensions are suspected4., 14., 15.. The investigation of choice is CT scan or MRI with the later being superior if cavernous sinus thrombosis is suspected.

Orbital complications include preseptal cellulitis, orbital cellulitis and abscess development. Preseptal cellulitis can be diagnosed clinically. It is an infection of the eyelid and surrounding skin anterior to the orbital septum that does not penetrate the periorbita. Orbital cellulitis occurs when infection spreads posterior to the orbital septum and can lead to abscess if not adequately treated. Radiological examination is required when orbital abscess is suspected^16^. Signs that indicate abscess formation include decrease in visual acuity, proptosis, ophthalmoplegia and pain associated with eye movement. Sometimes, it may be difficult to distinguish between preseptal and orbital involvement based on clinical observations alone especially with bilateral involvement. Such patients should be scanned and treated aggressively3., 17., 18..

In our study, the prevalence of orbital cellulitis was low (5.8%). The majority of the cases were children (72.2%). Most of the cases in children were preseptal cellulitis (21 out of 26 patients). Five patients (19.2% of children) had orbital cellulitis and no patient showed abscess. A total number of 10 patients were adults. Half of them had preseptal cellulitis, three patients (30%) had orbital cellulitis and two patients (20%) had abscess. These findings support that sinusitis related complications tend to occur in children but severe involvement are commoner in adults4., 7., 13.. Table 1 showed that orbital cellulitis and abscess formation were more statistically significant in adults compared to children (*p* < 0.05).

The distinction between preseptal, orbital cellulitis and abscess is important as it affects management strategy. Preseptal cellulitis can be treated on outpatient basis with oral and topical antibiotics. Orbital cellulitis requires hospitalization and intravenous antibiotics and orbital abscess may require surgical drainage. The aim of surgery is to decompress the orbit, drain an abscess or open infected sinuses. Delay in treatment may result in vision threatening complications^19^. The severe morbidity and mortality may result from intracranial complications including meningitis, cavernous sinus thrombosis and cerebral abscess. In our series, all patients showed complete recovery with treatment. All patients with preseptal cellulitis responded to local and oral treatment and none of them were hospitalized. Admission and intravenous antibiotics were required in 10 patients (those who had orbital cellulitis or abscess). Surgery was done only for one patient who needed drainage of sub periosteal abscess.

## CONCLUSION

Orbital complications are not frequent in patients with sinusitis. They are more common in children than adults. The prognosis is favorable; however, they can lead to devastating complications if not properly treated.
